# Improving porcine *in vitro* fertilization output by simulating the oviductal environment

**DOI:** 10.1038/srep43616

**Published:** 2017-03-06

**Authors:** Cristina Soriano-Úbeda, Francisco A. García-Vázquez, Jon Romero-Aguirregomezcorta, Carmen Matás

**Affiliations:** 1 Department of Physiology, Faculty of Veterinary Science, International Excellence Campus for Higher Education and Research “Campus Mare Nostrum”, University of Murcia, Murcia, Spain; 2 Institute for Biomedical Research of Murcia (IMIB-Arrixaca), Murcia, Spain; 3 Department of Animal Production, Faculty of Veterinary Science, University CEU Cardenal Herrera, Alfara del Patriarca, Valencia, Spain

## Abstract

Differences between the *in vitro* and *in vivo* environment in which fertilization occurs seem to play a key role in the low efficiency of porcine *in vitro* fertilization (IVF). This work proposes an IVF system based on the *in vivo* oviductal periovulatory environment. The combined use of an IVF medium at the pH found in the oviduct in the periovulatory stage (pH_e_ 8.0), a mixture of oviductal components (cumulus-oocyte complex secretions, follicular fluid and oviductal periovulatory fluid, OFCM) and a device that interposes a physical barrier between gametes (an inverted screw cap of a Falcon tube, S) was compared with the classical system at pH_e_ 7.4, in a 4-well multidish (W) lacking oviduct biological components. The results showed that the new IVF system reduced polyspermy and increased the final efficiency by more than 48%. This higher efficiency seems to be a direct consequence of a reduced sperm motility and lower capacitating status and it could be related to the action of OFCM components over gametes and to the increase in the sperm intracellular pH (pH_i_) caused by the higher pH_e_ used. In conclusion, a medium at pH 8.0 supplemented with OFCM reduces polyspermy and improves porcine IVF output.

In pigs, unlike other species, the efficiency of *in vitro* embryo production is very low because of the high incidence of polyspermy that occurs during *in vitro* fertilization (IVF)^1^. Such polyspermic fertilization might be related with the high number of spermatozoa required to attain an acceptable penetration rate compared with the number that reaches the oviduct *in vivo*. Another probable cause is that spermatozoa capacitation *in vitro* is not a sequential process that provides capacitated and partially reacted spermatozoa around fertilization time^2^ as occurs *in vivo*. Numerous IVF systems have been developed using different spermatozoa capacitation methods^3^,^4^, fertilization media^5^ or IVF devices^1^. However, despite the improvements achieved, there is no efficient and replicable method that can be used easily in IVF laboratories.

Under *in vivo* conditions, millions of spermatozoa are deposited in the female genital tract, but only a small subpopulation will reach the oviduct and site of fertilization. In the oviduct, spermatozoa bind to the epithelium and form the reservoir, where they will remain in a state of low-activity or uncapacitated status^2^. When ovulation occurs, spermatozoa separate from the epithelium and swim toward the ampullary region, where fertilization takes place^6^. For this purpose, spermatozoa acquire a type of special movement named hyperactivation, which is characterized by a high amplitude and an asymmetrical tail bending behaviour which results in a significantly higher swimming force than normal motility^6^.

Mammalian sperm encounter an acidic medium with a low HCO_3_^−^ concentration in the epididymis, which keeps them quiescent, and in the female the pH changes from acid in the vagina to basic in the oviduct, where the luminal fluid has a high concentration of HCO_3_^−^, the increasingly alkaline condition being necessary for fertilization^7^. The pH of the oviductal lumen, due to the production of HCO_3_^−^ in the epithelial cells lining it, greatly increases upon ovulation, and reaching a pH of around 8.0 in the oviductal ampulla in the periovulatory phase in of porcine^2^.

Spermatozoa intracellular pH (pH_i_) is a key regulator of spermatozoa motility and fertilizing ability^8^. It should be noted that several unique sperm ion transporters and enzymes, whose absence causes infertility, are either pH_i_ dependent or in some way related to pH_i_ regulation^9^. It has been shown that a reduction in [H^+^]_i_ and an increase vin [Ca^2+^]_i_ are required for the hyperactive movement, which is controlled by the opening of H^+^ and Ca^2+^ channels in the spermatozoa membrane^8^. These channels are in part regulated by the progesterone (P4) released by the ovaries and the cumulus cells surrounding the egg, by the glycoproteins of the ZP and by albumin, the main protein of the oviductal fluid (OF)^10^. The hyperactive movement allows spermatozoa to exit the epithelium folds, swim through the viscous OF and penetrate the egg vestments. However, less than 20% of the spermatozoa population develops this kind of movement^11^.

Once the spermatozoa are released from the reservoir they must find the oocyte, but the chances of such a low number of spermatozoa successfully reaching the egg without a guidance mechanism are very slim. For this reason, it is believed that spermatozoa must be guided along the oviduct in order to reach the oocyte. Indeed, it seems that spermatozoa are equipped with a mechanism for moving towards the oocyte in response to thermotaxis, rheotaxis and chemotaxis, each depending on a specific stimulus: a temperature gradient, fluid flow and a chemoattractant gradient, respectively^12^.

Several substances that act as spermatozoa attractants *in vivo* have been identified within the follicular fluid (FF) and OF, while others are secreted by cumulus cells and the oocyte (COCs). Among these molecules are P4, which is considered the main chemoattractant, nitric oxide^13^, adenosine^14^, hyaluronic acid (HA)^15^ and glycodelin^16^. Spermatozoa chemoattractants, which are not only secreted prior to ovulation within the follicle, but also after oocyte maturation outside the same, have two origins: the mature oocyte and the surrounding cumulus cells^17^.

In *in vitro* conditions the preincubation of oocytes in OF has been demonstrated as a good method for increasing IVF efficiency in pigs, as the ZP is modified through the binding of specific components from the OF^18^. It has also been shown that OF contains P4^19^ and improves embryo quality^20^.

In order to improve IVF efficiency in pig, we have designed a system based on some of these *in vivo* conditions. Spermatozoa were physically separated from the oocyte into an alkaline medium supplemented with OF and FF, where the capacitation process could start. After capacitation, the sperm population, it was thought, would be able to respond to the attractants released by COCs and, consequently, swim toward the oocyte and fertilize it.

## Results

### Experiment 1: Effect of pH_e_, device and OFCM on *in vitro* fertilization (IVF)

#### IVF

During fertilization the environment in which gamete interaction takes place has a pH close to 8.0^2^,^21^ but this encounter of gametes does not occur by a random process: rather, the spermatozoa must be guided towards the oocyte by different mechanisms^12^ through the tortuous path that is the isthmus.

So, this experiment was interested to simulate the *in vivo* fertilization conditions, studying the effect of increasing pH_e_ (from 7.4 to 8.0), adding OFCM (formed by FF, OF and COCs secretions) and separating the gametes using an S device in which the spermatozoa face a barrier and are physically far from the oocytes (simulating isthmus-ampulla conditions). The IVF results for penetration, monospermy and efficiency are shown in Table 1, where they are expressed as percentages.

The pH increase in the IVF medium and the addition of OFCM affected all three studied parameters (P < 0.05). Although penetration rates were lower when pH_e_ 8.0 was used, monospermy was higher, meaning higher efficiency. However, using the S device did not affect any of the three parameters studied (P > 0.05). The addition of OFCM to IVF only affected the penetration results when pH_e_ 7.4 and the W device were used, penetration in this case decreasing from 76.7% to 58.8%. Monospermy and efficiency rates increased in the presence of OFCM, reaching the highest percentages at pH_e_ 8.0 with the S device. In short, the use of pH_e_ 8.0 and OFCM in an S device resulted in the highest efficiency (putative zygotes) (48.7 ± 4.7%).

#### ZP solubility

It has been established that the ZP of oocytes matured *in vivo* is highly resistant to pronase digestion^22^. This hardening may be the result of the deposition of oviductal secretions and is reversible^18^. However, it is not known if pH_e_ affects ZP solubility. Therefore, we measured ZP solubility under different conditions.

**Figure 1:**
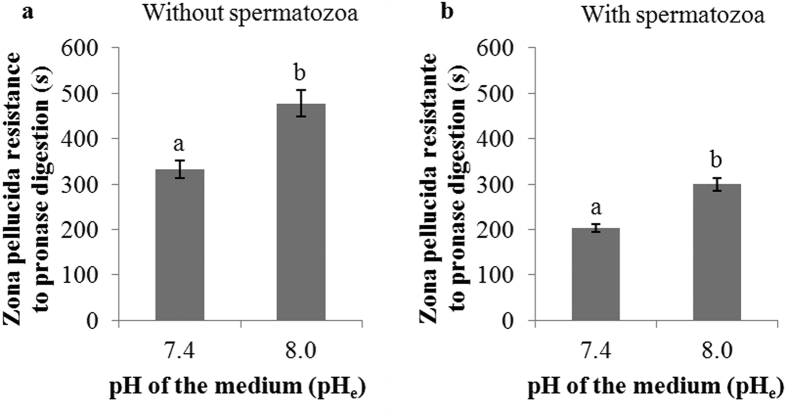
Zona pellucida resistance to pronase digestion in pig oocytes incubated for 180 min in TALP medium at two pH values (7.4 and 8.0). (**a**) Without spermatozoa co-culture. (**b**) With spermatozoa co-culture. Results expressed as mean ± SEM. Different letters (**a,b**) show statistical differences (P < 0.05).

ZP resistance to pronase digestion was significantly higher (P < 0.05) in oocytes incubated at pH_e_ 8.0 than at pH_e_ 7.4, taking 477.8 ± 30.1 and 332.4 ± 20.3 s, respectively, to dissolve (Fig. 1a). In the presence of spermatozoa, pH_e_-dependent differences were noted: ZP resistance after the co-culture of oocytes and spermatozoa at pH_e_ 7.4 was significantly lower than at pH_e_ 8.0 (203.6 ± 8.9 *vs.* 299.0 ± 14.0 s, P < 0.05) (Fig. 1b). The results from the present study indicate that pH_e_ affects ZP solubility both in the presence and absence of spermatozoa. Shorter dissolution times were recorded when the pH_e_ used was 7.4, with or without the presence of spermatozoa.

#### P4 concentration

The effects of P4 on spermatozoa include inducing acrosome reaction (AR), attracting the spermatozoa and/or modulating their hyperactivation in a dose-dependent manner. When P4 concentration was checked in FF (n = 6), OF (n = 5) and CM (n = 7), the results were 36.9 ± 0.5, 54.0 ± 2.4 and 105.1 ± 4.5 ng/ml (mean ± SEM), respectively. So, P4 concentration in OFCM (1% OF and 2% CM), where spermatozoa were incubated to assess functionality, was 2.6 ± 0.2 ng/ml.

### Experiment 2: Effect of pH_e_ and OFCM on spermatozoa functionality and pH_i_

#### Spermatozoa functionality

In *in vivo* conditions and before gamete encounter, sperm are exposed to different environments in preparation for fertilization. In experiment 1 it was noticed that the presence of pH_e_ 8.0 and oviductal substances modified IVF output, so the effect of these factors on sperm functionality was investigated. The samples were incubated in a capacitating medium at pH 7.4 or 8.0 and in the presence or absence of OFCM. Several parameters of spermatozoa functionality were determined at different times of incubation.

##### Spermatozoa motility

When ovulation occurs, spermatozoa are released from the sperm reservoir before they plunge into the oviduct and swim to the fertilization site at the ampullary-isthmic junction^23^. During this process, the oviductal environment prepares the spermatozoa for fertilization, and, among other events, the oviductal pH increases and the motility of the spermatozoa increases (hyperactivation)^24^. This experiment studied the effect that such a periovulatory environment (pH_e_ 8.0 and the presence of OFCM) has on spermatozoa motility, as evaluated by CASA (Table 2). All the motility parameters studied were affected by pH_e_ (P < 0.05) except MotPro and WOB. In general terms, pH_e_ 8.0 produced lower motility parameters than pH_e_ 7.4, with lower velocities (VCL, VSL and VAP), linearity (LIN), straightness (STR), amplitude of lateral head displacement (ALH) and beat cross-frequency (BCF). When OFCM was added, the motility pattern was similar to that just described or even lower since all the motility parameters except WOB were affected by its addition. The combined use of pH_e_ 8.0 and OFCM (8.0OFCM) affected all motility parameters which were all significantly reduced (P < 0.05) compared with the 7.4 group, except WOB.

##### Western blotting (WB): Protein Kinase A substrates and Tyrosine Phosphorylation (PKAs-P and Tyr-P)

**Figure 2:**
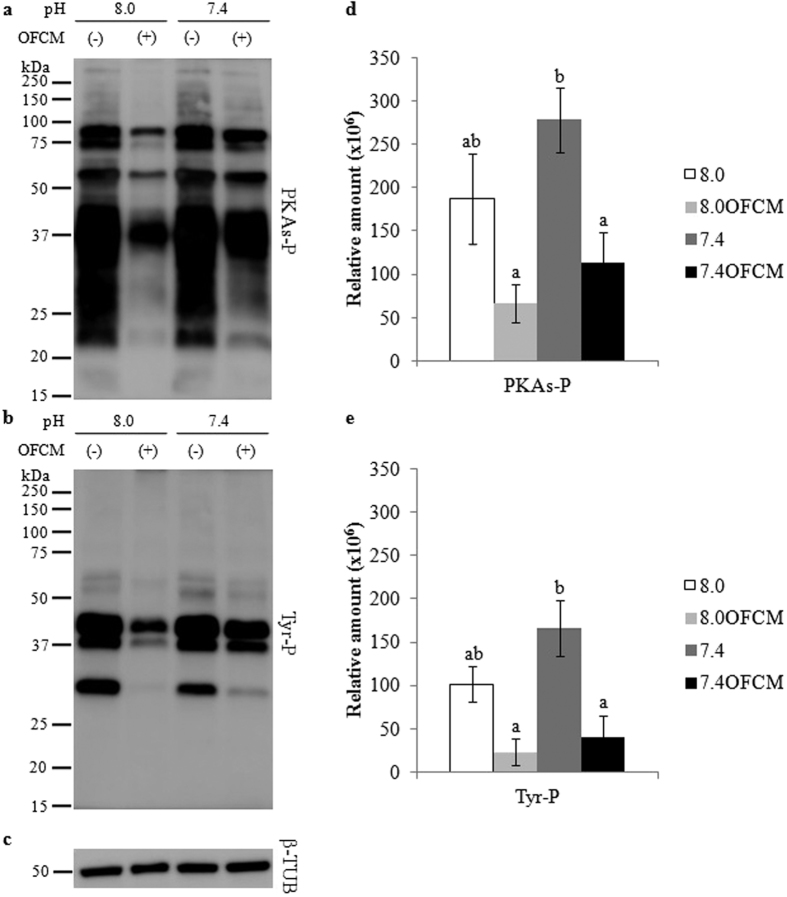
Effect of pH_e_ and OFCM on both PKA substrates and Tyrosine phosphorylation (PKAs-P and Tyr-P). Sperm were incubated for 180 min under capacitating conditions using both different pH_e_ values (7.4 and 8.0) and in the presence (or not) of OFCM. (**a** and **b**) Sperm protein extracts were analyzed for phosphorylation by western blotting using α-pPKAs or α-pTyr as first antibodies, respectively. (**c**) Loading control with β-tubulin (β-TUB). (**d** and **e**) Relative amount of signal quantified in each membrane using ImageQuant TL v8.1 software for PKAs-P and Tyr-P, respectively. Different letters (**a,b**) indicate statistically significant differences (P < 0.05) between groups.

Spermatozoa need to undergo a series of changes before they can fertilize, in a process known as capacitation. This phenomenon involves the early activation of protein kinases and the inactivation of protein phosphatases. To investigate the effect of the oviductal periovulatory environment (pH_e_ 8.0 and presence of OFCM) on the *in vitro* spermatozoa capacitation process, PKAs-P and Tyr-P were analyzed and quantified by WB (Fig. 2) since they are indicative of spermatozoa capacitation status^25^.

The WB for PKAs-P showed the same degree of phosphorylation in spermatozoa incubated at pH_e_ 7.4 and 8.0. However, when spermatozoa were incubated with OFCM, the signal intensity was lower than without it (Fig. 2a and d). In the same way, the results showed that increasing pH_e_ is not necessarily associated with an increase or decrease in Tyr-P, while adding substances such as OFCM leads to a significantly lower degree of activation of this protein or a lower number of spermatozoa with Tyr-P (Fig. 2b and e).

##### Immunocytochemistry: Tyrosine Phosphorylation (Tyr-P) observed by Indirect Immunofluorescence (IIF)

**Figure 3:**
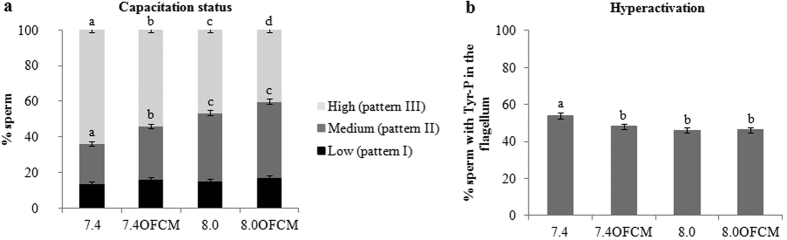
Effect of pH_e_ and OFCM on sperm capacitation status according to immunolocation of protein tyrosine phosphorylation (Tyr-P). Spermatozoa were incubated for 180 min under capacitating conditions using both different pH_e_ values (7.4 and 8.0) and in the presence (or not) of OFCM. Results are shown as mean ± SEM. Different letters (**a–d**) in the same pattern of each column indicate statistically significant differences (P < 0.05). (**a**) Percentage of sperm with low capacitation status (pattern I: non-phosphorylated or head- and/or flagellum-phosphorylated spermatozoa), medium capacitation status (pattern II: equatorial segment or equatorial segment and flagellum phosphorylated) or high capacitation status (pattern III: equatorial segment and head and/or flagellum phosphorylated). (**b**) Percentage of hyperactivated sperm (pattern IV: flagellum phosphorylated regardless in other locations).

Tyr-P localization in the spermatozoa is related to their degree of capacitation (capacitation status)^26^. In this experiment, the immunolocation of Tyr-P was analyzed by IIF, which allowed different spermatozoa subpopulations to be identified within a sample according to their degree of capacitation and hyperactivation (Fig. 3a and b).

There were no statistical differences between groups as regard the percentage of sperm with a low capacitation status (pattern I, P > 0.05). In the case of pattern II (considered as medium capacitation status), it was observed that at pH_e_ 8.0 the number of sperm showing this pattern increased. It was also recorded that when pattern II increased, pattern III (considered as high capacitation status) decreased, especially with the addition of OFCM. This means that pH_e_ 8.0 maintains spermatozoa in a state of low capacitation, especially in the presence of OFCM.

Figure 3b shows the Tyr-P in the flagellum, independently of other locations related with sperm hypermotility (hyperactivation pattern). According to the motion parameters described above, the 7.4 group had the highest rate of Tyr-P in the flagellum (P < 0.05).

##### Acrosome Reaction (AR)

**Figure 4:**
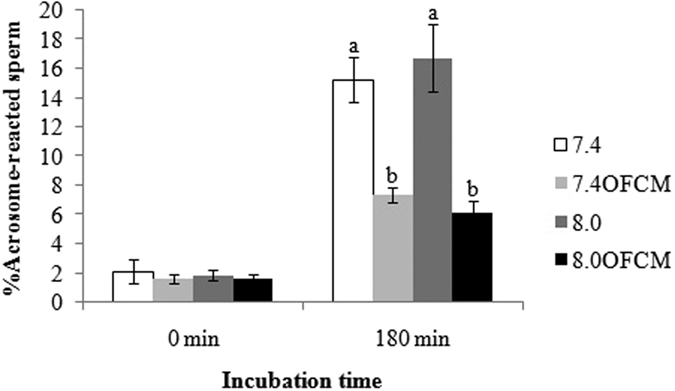
Effect of pH_e_ and OFCM on sperm acrosome reaction (AR). Spermatozoa were incubated for 0 and 180 min in capacitating medium using both different pH_e_ values (7.4 and 8.0) and in the presence (or not) of OFCM. Different letters (**a,b**) in the same time of incubation indicate statistically significant differences (P < 0.05).

Following spermatozoa capacitation, the AR takes place in response to an agonist such as P4 or ZP^27^. *In vivo*, the oviductal environment to which spermatozoa are exposed protects the spermatozoa from a premature AR^28^,^29^, which the spermatozoa only undergo when in contact with the oocyte. Therefore, this experiment studied the *in vitro* effect of pH_e_ 8.0 and the presence of OFCM on spermatozoa AR at 0 and 180 min of incubation (Fig. 4).

At the beginning of incubation (0 min), as expected, all groups showed a low proportion of acrosome-reacted spermatozoa with no significant differences between them (P > 0.05). After 180 min of incubation in the medium, the proportion of acrosome-reacted spermatozoa had increased in all groups but in different ways. The pH_e_ had no statistically relevant effect but the addition of OFCM made a great difference considerably lowering the percentage of acrosome-reacted sperm.

#### *Spermatozoa pH*_*i*_

During fertilization process spermatozoa are exposed to a varying pH_e_ and capacitation is associated to an intracellular alkalinisation in sperm of many mammalian species. On the other hand, the metabolic activity acidifies the cytosol and changes in pH_i_ affect the ionization state of the weak acids and bases present in most proteins and many biomolecules. For the reason explained above, it was decided to analyse whether OFCM modified the pH_i_ when the pH_e_ increased.

**Figure 5:**
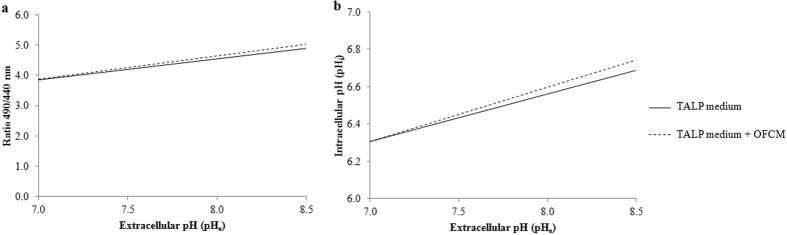
Effect of extracellular pH (pH_e_) and OFCM on intracellular pH (pH_i_) in boar spermatozoa incubated in TALP medium. (**a**) Fluorescence emission ratio with BCECF-AM when samples were exposed to different pH_e_ values in a range from 7.0 to 8.5 in the absence or presence of OFCM and excited at 490 and 440 nm. (**b**) pH_i_ calculated from the emission ratio (490/440 nm) when spermatozoa are exposed to different pH_e_ in a range from 7.0 to 8.5 in absence or presence of OFCM.

The results showed the fluorescence ratio at 490/440 nm and the pH_i_ when spermatozoa were exposed to different pH_e_ (Fig. 5a and b). The spermatozoa had a lower pH_i_ than pH_e_ but they were correlated (r = 0.954) since when pH_e_ increased, the pH_i_ increased too. However, for all values of pH_e_ studied the presence of OFCM had no effect on pH_i_ (P = 0.752).

## Discussion

The current *in vitro* production of potentially viable porcine embryos is not efficient mainly due to the high incidence of polyspermy^1^ and it is clear that the microenvironment in which fertilization occurs plays a key role^18^,^30^. The present study attempts to improve previous results by ensuring the presence of conditioned medium [COCs secretions, oviductal and follicular fluids (OFCM)], using an IVF device in which spermatozoa are physically separated from the oocytes, but in which they can swim towards them, and, finally, the exposing the gametes to specific oviductal factors at different pH values.

The pH in the porcine oviductal ampulla is close to 8.0 during the periovulatory phase^2^. For this reason, the first factor analyzed in the present study was the effect of increasing the pH_e_ from 7.4 (current IVF system) to 8.0. The IVF results showed that efficiency was higher at pH_e_ 8.0, which seems reasonable because most biological processes that occur in living organisms are regulated by the pH level^31^ and fertilization is no exception. The slight increase in pH_e_ may be acting in two different ways to decrease polyspermy. On the one hand, it could be slowing down the enzymatic activity of the acrosomal content over ZP proteins, which would reduce the number of spermatozoa that completely dissolve the ZP proteins in the short time available for fertilization, resulting in a protective effect against multiple penetrations. On the other hand, pH_e_ 8.0 could be improving the release of cortical granules to the perivitelline space, promoting the correct conformational changes of ZP proteins that prevent polyspermic penetration^5^,^32^. In order to test this possibility, a ZP hardening assay was performed. The results showed that ZP resistance to dissolution was higher in oocytes incubated at pH_e_ 8.0 after culture both in the presence and in absence of spermatozoa. Nonetheless, the effect of pH_e_ on ZP hardening was not comparable to the effect of using periovulatory OF^14^.

Porcine IVF is usually carried out without barriers between the spermatozoa and oocytes and with a high number of spermatozoa per oocyte. Therefore, the *in vitro* penetration of oocytes is more a matter of coincidence than a competition and selection process that occurs *in vivo*^33^. In IVF systems, each oocyte has contact with many spermatozoa at the same time. Besides, it has been shown that the presence of a large number of spermatozoa at an early stage of the spontaneous AR in co-culture^34^ results in high polyspermy rates. Therefore, using a device in which gametes are separated and which spermatozoa have to swim up to find oocytes could be considered a good way to improve monospermy. When we used an S device, as used in^34^, neither monospermy nor efficiency improved. However, these authors used frozen semen, which could mean limited sperm survival. In the present work, fresh semen was used, and it is known that the corresponding spermatozoa can penetrate oocytes for up to 23 h of co-culture^35^, enabling spermatozoa to undergo capacitation and swim toward the oocyte for a longer time.

The third condition that could improve efficiency was to mimic the *in vivo* fertilization environment by using secretions produced in the oviduct during the periovulatory stage. So, OFCM medium was added to the IVF system. Our results showed that components of the OFCM seemed to be acting on the gametes and/or their interaction, although this effect was only relevant when IVF was performed at pH_e_ 7.4. This result agrees with those of other authors, who reported the hardening effect of OF on ZP and the consequent prevention of multiple penetration^18^. When a pH_e_ of 8.0 was used, the addition of OFCM did not increase the efficiency. Perhaps pH_e_ 8.0 in itself is enough to improve the IVF output, although it has been shown that OF has any beneficial effect on subsequent embryo quality^20^.

Spermatozoa motility, PKA activity, Tyr-P and AR were analysed in order to clarify whether the IVF results described above could be attributed to the spermatozoa capacitation status. As has been reported, the pattern of spermatozoa movement varies among species and with the physical environment in which the spermatozoa swim. However in mammals the motility of recently ejaculated spermatozoa is characterized by a relatively low-amplitude and symmetrical tail bending, but, when hyperactivated, this motility changes to a high-amplitude accompanied by asymmetrical tail bending (observed close to the site of fertilization)^6^. The results of our study showed that pH_e_ 8.0 with OFCM led to a decrease in all motility parameters except for WOB although the main factor responsible was the addition of OFCM. Some authors^36^ observed that a pH_e_ below 7.2 affected sperm motility and capacitation but there was no effect between pH_e_ 7.2 and 8.2. This apparent inconsistency with our results could be explained by the different experimental conditions used in the studies, the sperm source (human sperm *vs*. porcine sperm) and the incubation times.

When spermatozoa were incubated with OFCM, the values of all motility parameters decreased, perhaps because this medium reverses the influence of the capacitating factors present in TALP medium. Besides, it has been shown that the pattern of hyperactivation is greatly influenced by both the surrounding microstructure and the viscosity of the medium^37^,^38^. It is known that certain components of OFCM medium, such as P4, are chemoattractants and produce lower hyperactivation^39^. OF and HA prolong spermatozoa survival and delay destabilization processes at the plasmalemma i.e. spermatozoa capacitation^40^ and resulting hyperactivation. In fact, the sperm reservoir regulates the physiological status of the spermatozoa, particularly the processes of capacitation and hyperactivation within the SR to ensure that spermatozoa are in the proper state when ovulation occurs^41^. The other factor to consider is the medium’s viscosity since viscoelastic fluids can reduce the swimming velocity of spermatozoa^38^. The OFCM medium is more viscous than TALP^30^, and an increase in the viscoelasticity has been associated with a lower percentage of motile spermatozoa as well as with lower VCL, VAP and ALH^42^. Some authors^30^,^43^ also showed that viscosity affected the spermatozoa motility parameters but the results varied depending on the degree of the viscosity of the medium.

Capacitation status has been associated with an increase in PKA activity and phosphorylation in tyrosine residues of general sperm proteins^44^. Although increasing the pH_e_ to 8.0 provoked an increase of 0.2 points in pH_i_, it seemed to have no significant effect on sperm capacitation and pH_e_ 7.4 is enough to reach a high grade of PKAs-P and Tyr-P. However, pH_e_ 8.0 had a significant effect, increasing ZP resistance to digestion and probably playing a key role in gametes interaction. In the present study, we showed that the presence of OFCM in the capacitation medium decreased PKA activity and the Tyr-P of sperm proteins, suggesting that OFCM interferes with the cyclic AMP (cAMP)/PKA/Tyr-P pathway but in a way that is still unknown. In this same sense, the localization of Tyr-P showed that the presence of OFCM produced a lower percentage of highly capacitated spermatozoa, especially at pH_e_ 8.0, supporting the idea that their components could act by preserving their function and fertilizing ability.

In *in vivo* conditions, capacitation is inhibited while the spermatozoa remain in the oviductal reservoir^45^,^28^ despite the abundant presence of bicarbonate (35–90 mM)^2^ which would normally be sufficient to induce capacitation and rapid flagellar activity *in vitro*. Apart from bicarbonate-induced effects during sperm capacitation, it is also important to notice how oviduct proteins and fluids may affect the composition and organization of proteins on the sperm surface^46^, acting to sequentially activate different signalling pathways. It is known that the adenosine present in the FF regulates the adenylyl cyclase (AC)/cAMP signal transduction pathway stimulating cAMP production in uncapacitated cells but inhibiting it in capacitated ones^14^. The hyaluronic acid (HA) from the OF and FF present in the oviduct can modulate sperm capacitation and promote sperm survival^2^, and HA from cumulus cells promote the AR close to the oocyte^47^. Besides, it has been suggested, some oviductal proteins could be involved in modulating sperm function and fertilization^28^; for example, Glycodelin-A, -S, -F and -C bind to the sperm head during their passage through the female genital tract and first act by inhibiting the sperm-zona pellucida binding and suppressing the P4-induced AR and finally stimulate sperm-zona pellucida binding^16^. Additionally, the OF contains soluble proteins such as oviduct glutathione peroxidase, superoxide dismutase and catalase, that may regulate the balance between reactive oxygen species and antioxidants in sperm -which is important for sperm viability and motility^48^- and so regulate the capacitation process^49^.

The oviduct environment acts as the sperm reservoir, maintaining spermatozoa viability and modulating the subpopulation of spermatozoa that initiates the capacitation process, preventing a premature AR and resulting in a lower rate of polyspermy, as previously described^28^,^29^. The results of the present study showed that OFCM components decrease the induced AR after incubation of the spermatozoa in capacitation conditions and maintain their fertilizing potential probably via AC/cAMP, as has been previously reported^14^. This could be attributed to a decrease in PKA activation and Tyr-P. It also has been determined that PKA, together with inositol-trisphosphate, activates Ca^2+^ channels in the outer acrosomal membrane, which leads to an increase in cytosolic Ca^2+^. The depletion of Ca^2+^ in the acrosome will activate a store-operated Ca^2+^ entry mechanism in the plasma membrane, leading to increase in cytosolic Ca^2+^ and resulting in membrane fusion and AR^50^. Therefore, the effect of OFCM on PKA activity and AR might help to explain the decrease in polyspermy percentage observed. P4, another component of OFCM, induces AR but this effect seems to be prevented by adenosine and glycodelins in OFCM^16^. The low concentration of P4 detected in OFCM (much lower concentration than that used for *in vitro* AR induction^51^) was probably responsible for guiding the spermatozoa to the oocytes but did not produce a higher level of capacitation or induce the AR^52^. However, the proteins of the OFCM could have been responsible for decreasing the number of P4 receptors in the sperm membrane, as has been suggested^53^.

Therefore, the main effect on spermatozoa of the substances present in OFCM seems to be keeping them in a state of low capacitation, thus regulating the number of spermatozoa ready for fertilization, leading to an increase in efficiency.

In conclusion, the results obtained in this work suggest that the IVF conditions proposed are more similar to the *in vivo* periovulatory environment in the oviduct than can be obtained with the currently used IVF protocol. Since the success of fertilization is a multifactorial process that is difficult to control *in vitro*, setting several periovulatory oviductal factors (presence of cumulus cells, follicular and oviductal fluids and adjusted pH) reduces polyspermy and increases IVF efficiency in pig. Manipulation of these components has enabled us to propose a new way of performing IVF, resulting in a greater number of potentially viable zygotes. However, more studies are necessary to clarify the improvements and to identify new conditions that might contribute to a better understanding of swine reproductive physiology.

## Material and Methods

### Ethics

The study was carried out following the Spanish Policy for Animal Protection RD 53/2013, which meets European Union Directive 2010/63/UE on animal protection. All the procedures carried out in this work have been approved by the Ethical Committee of Animal Experimentation of the University of Murcia and by the Animal Production Service of the Agriculture Department of the Region of Murcia (Spain) (ref. no. A13160609).

### *In vitro* maturation of oocytes (IVM)

COCs were collected from antral follicles (3–6 mm diameter) and washed with Dulbecco’s PBS (DPBS). Only COCs with complete and dense cumulus oophorus, in groups of 50, were cultured for 42 h in 500 μl NCSU-37, as previously described^54^.

### Conditioned medium (CM), follicular fluid (FF) and periovulatory oviductal fluid (OF) collection

After maturation, COCs were pipetted to mix their secretions with the surrounding NCSU-37 medium. The whole content of the wells was collected and centrifuged at 7,000 g for 10 min at 4 °C, discarding the oocytes and cumulus cells to obtain the supernatant (CM). The FF was collected from antral follicles (3–6 mm diameter) of prepuberal gilts, as previously described^55^. The OF was obtained from a pool of porcine oviducts with ovaries close to ovulation, as described^56^. The FF and OF were centrifuged as described for CM. The CM, FF and OF were aliquoted and stored at −20 °C until use.

### Sperm collection

The sperm-rich fraction was collected from boars with proven fertility by the manual method. Sperm concentration, motility, acrosome integrity, and normal morphology were microscopically evaluated by standard laboratory techniques.

### *In Vitro* Fertilization (IVF)

**Figure 6:**
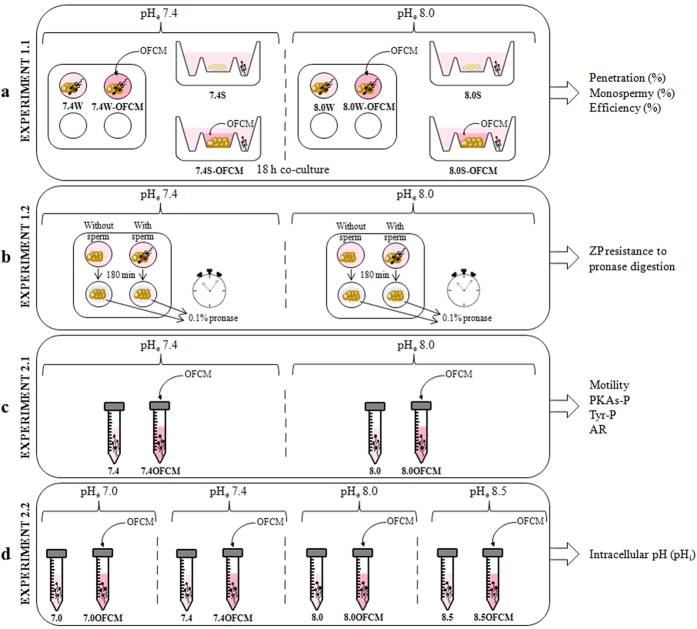
Experimental design. (**a**) Experiment 1.1: analysis of the effect of pH_e_ (7.4; 8.0), device (W: 4-well multi-dish; S: inverted screw cap of a conical tube) and OFCM (1% OF and 2% CM) on IVF parameters (percentages of penetration, monospermy and efficiency). (**b**) Experiment 1.2: analysis of the effect of pH_e_ on zona pellucida solubility after 180 min of incubation with or without spermatozoa. (**c**) Experiment 2.1: assessment of the effect of pH_e_ and OFCM addition on spermatozoa functionality: motility, tyrosine phosphorylation (Tyr-P), protein kinase A substrates phosphorylation (PKAs-P) and acrosome reaction (AR). (**d**) Experiment 2.2: assessment of the effect of pH_e_ and OFCM addition on spermatozoa pH_i_.

The medium used for IVF was Tyrode’s albumin lactate pyruvate (TALP)^57^ (supplemented or not with OFCM) equilibrated for almost 3 h at 38.5 °C and under 5 or 1.5% of CO_2_ to adjust the pH of the medium to 7.4 or 8.0, respectively (according to the Henderson-Hasselbalch equation). IVF was performed with 2 types of device: (i) a 4-well multi-dish containing 500 μl TALP per well (W)^58^, and (ii) inverted screw cap of a conical centrifuge tube (Falcon^®^ 15 ml high-clarity polypropylene conical centrifuge tubes with polyethylene dome seal screw caps) containing 1000 μl TALP (S). For a full description of the device see^59^. After IVM, the oocytes (with or without cumulus cells) were gently deposited in W or in the inner plate of the S device, depending on the experimental group (Fig. 6). The sperm cells were added to W or to the outer plate of the S device to give a final concentration of 1 × 10^5^ cells/ml. After 18 h of co-culture the putative zygotes were fixed and evaluated as previously described^4^.

### Assessment of zona pellucida (ZP) solubility

The IVM oocytes, before or after IVF, were washed by pipetting to remove surrounding cells and added to 100 μl of 0.1% (w/v) pronase solution in PBS. ZPs were continuously observed for dissolution under an inverted microscope equipped with a warm plate at 37 °C^5^. The ZP dissolution time of each oocyte was registered as the time elapsing between placing the samples in the pronase solution and the time when the ZP was no longer visible at a magnification of x200.

### Chemiluminiscent microparticle immunoassay (CMIA)

The P4 concentration in FF, OF and CM was assessed by chemiluminiscent microparticle immunoassay (CMIA; Architect, Abbott), as described^19^.

### Spermatozoa motion assay

Computer-assisted spermatozoa motility analysis (CASA) was performed using ISAS^®^ system (PROISER R + D S.L., Valencia, Spain). For this propose, a 6 μl drop of the sample was placed on a warmed (38.5 °C) 20 micron Leja^®^ slide (SC-20–01–02-B, Leja Products B.V., Nieuw Vennep, The Netherlands) and was analyzed using a phase-contrast microscope (magnification x200; Leica DMR, Wetzlar, Germany).

### Western blotting (WB)

Isolated proteins from 1 × 10^6^ spermatozoa samples were obtained and immunoblotted as described^60^. The antibodies used were anti-protein kinase A (9624, Cell Signaling Technology, Beverly, USA, 1:2,000), anti-phosphotyrosine (4G10, Millipore, CA, USA, 1:10,000) and anti-β-tubulin (T0198, Sigma-Aldrich^®^, Madrid, Spain, 1:5,000). Blots were visualized by chemiluminescence (Amersham Imager 600, GE Healthcare) using a Pierce^®^ ECL 2 Western Blotting Substrate (80196, Lumigen Inc, Southfield, MI, USA). The relative amount of signal in each membrane was quantified using the ImageQuant TL v8.1 software (GE Healthcare, Life Sciences, Buckinghamshire, UK).

### Immunocytochemistry: Tyrosine Phosphorylation (Tyr-P) detection by Indirect Immunofluorescence (IIF)

IIF was carried out to study Tyr-P location as described^58^ using the same anti-phosphotyrosine as used for Tyr-P detection by WB (1:300 in BSA 1%). The secondary antibody used was fluorescein-conjugated goat anti-mouse (Bio-Rad Laboratories, Madrid, Spain, 1:400 in BSA 1%).

### Acrosome Reaction (AR) assay

The AR was assessed by staining with FITC-conjugated peanut agglutinin from *Arachis hypogaea* (PNA-FITC L7381, Sigma-Aldrich^®^, Madrid, Spain) as previously used^61^. Samples were analyzed under an epifluorescence microscope (blue filter, BP 480/40; emission BP 527/30; Leica^®^ DM4000 B LED, USA) at x400 magnification.

### Measurement of sperm pH_i_

Spermatozoa (30 × 10^6^ cells/ml) were loaded with 5 μM of the pH-sensitive dye BCECF-AM (B1150, Sigma-Aldrich^®^, Madrid, Spain) for 30 min incubation at 38.5 °C. Extracellular dye was removed by centrifugation at 700 g for 3 min. The spermatozoa were resuspended in PBS without Ca^2+^ and Mg^2+^ and incubated again for an additional 15 min at 38.5 °C to allow de-esterification of the dye. After that, the samples were centrifuged and resuspended in the corresponding medium, depending on the experimental group. The fluorescence was immediately monitored using a spectrofluorometer (FP-6300, Jasco^®^, Cremella, Italy). The system was first calibrated using BCECF-AM equilibrated spermatozoa at pH 7.0, 7.4, 8.0 and 8.5 in the presence of 0.1% Triton X-100 and adjusting the pH with HCl and NaOH^62^. The cells were excited at both 490 and 440 nm wavelength and the emission spectra were recorded at 535 nm. The emitted fluorescence ratio from the excitation at 490/440 nm was calculated and the regression line for pH_e_ *vs.* the 490/440 nm ratio was plotted. Finally, the pH_i_ values of the sperm cells was estimated from that regression line.

## Experimental Design

This study aimed to mimic several *in vivo* conditions during fertilization to improve IVF efficiency. In persuit of these conditions, we used TALP medium supplemented with OFCM, pH 8.0 and a device (S) which separated sperm and COCs. The OFCM was composed of OF (1%) and CM (2%). The OF concentration was based on previous studies^63^,^64^. However, the reasons to supplement with 2% CM were basically mechanical (because 50 COCs were taken in a volume of 20 μl) and to ensure that the P4 concentration was within the range of values that occur in the oviduct during fertilization^19^. The spermatozoa capacitation status after incubation at pH_e_ 8.0 and the addition of OFCM was also evaluated. For this purpose, two experiments were performed (Fig. 6).

### Experiment 1: Effect of pH_e_, device and medium supplementation on IVF

#### IVF

The IVF experimental groups were established depending on the pH of the TALP medium (7.4 or 8.0), the device used (W or S) and the presence or absence of OFCM as additive to the medium (Table 1). The experimental groups were: 7.4W (pH_e_ 7.4 and 4-well multidish), 8.0W (pH_e_ 8.0 and 4-well multidish), 7.4S (pH_e_ 7.4 and screw cap device), 8.0S (pH_e_ 8.0 and screw cap device), 7.4W-OFCM (pH_e_ 7.4, 4-well multidish and OFCM addition), 8.0W-OFCM (pH_e_ 8.0, 4-well multidish and OFCM addition), 7.4S-OFCM (pH_e_ 7.4, screw cap device and OFCM addition) and 8.0S-OFCM (pH_e_ 8.0, screw cap device and OFCM addition). A total of 1079 oocytes were used in 4 replicates to determine the percentage of penetrated oocytes (Penetration, %), monospermy percentage of penetrated oocytes (Monospermy, %) and efficiency (Efficiency, %), which represents the final number of putative zygotes in each group per 100 penetrated oocytes.

#### ZP solubility

*In vitro* matured oocytes incubated for 3 h in TALP (at pH 7.4 and 8.0) and oocytes collected 3 h post-IVF (at pH 7.4 and 8.0) were subjected to ZP solubility analysis. 20 oocytes/zygotes per experimental group were used (4 replicates).

### Experiment 2: Effect of pH_e_ and OFCM on spermatozoa functionality and pH_i_

#### Spermatozoa functionality

Spermatozoa were incubated in TALP medium at pH 7.4 or 8.0 and supplemented or not with OFCM. Therefore, the experimental groups were: 7.4: pH_e_ 7.4 and non-supplemented TALP; 7.4OFCM: pH_e_ 7.4 and TALP supplemented with OFCM; 8.0: pH_e_ 8.0 and non-supplemented TALP; 8.0OFCM: pH_e_ 8.0 and TALP supplemented with OFCM. Several parameters of spermatozoa functionality were determined at different times of incubation.

##### Spermatozoa motility

Spermatozoa motility was determined at 30 min of incubation (4 replicates) in 3 different fields per sample. The parameters observed were the percentage of total motile spermatozoa (Mot, %), motile progressive spermatozoa (MotPro, %), curvilinear velocity (VCL, μm/s), straight line velocity (VSL, μm/s), average path velocity (VAP, μm/s), linearity of the curvilinear trajectory (LIN, ratio of VSL/VCL, %), straightness (STR, ratio of VSL/VAP, %), amplitude of lateral head displacement (ALH, µm), wobble of the curvilinear trajectory (WOB, ratio of VAP/VCL, %), and beat cross-frequency (BCF, Hz). Western blotting (WB): Protein Kinase A substrates and Tyrosine Phosphorylation (PKAs-P and Tyr-P).

After 180 min of incubation, PKAs-P and Tyr-P were determined by WB and semi-quantified in each experimental group in 4 replicates. β-tubulin (β-TUB) was used as loading control.

##### Immunocytochemistry: Tyrosine Phosphorylation (Tyr-P) by Indirect Immunofluorescence (IIF)

The Tyr-P location in the spermatozoa was evaluated by IIF after 180 min incubation. Two-hundred spermatozoa per sample were analyzed in 4 replicates. Then, the Tyr-P location of the spermatozoa proteins was classified and grouped into three different categories according to the patterns given by Luño *et al*.^26^: Pattern I (low capacitation) includes spermatozoa without fluorescence, those with phosphorylated acrosome, those with phosphorylated tail and those with phosphorylated acrosome and tail. Pattern II (medium capacitation) includes spermatozoa with fluorescence in the equatorial subsegment with or without the presence of signal in the flagellum. Pattern III (high capacitation) includes spermatozoa with signal in the equatorial subsegment and acrosome area and with or without the presence of signal in the flagellum. In addition, a fourth pattern was established related with the hypermotility capacity, which included those spermatozoa with a signal in the flagellum regardless of other locations (hyperactivation pattern).

##### Acrosome Reaction (AR)

The AR was analyzed at 0 and 180 min of incubation in 4 replicates. Two-hundred spermatozoa per sample were analyzed and classified as reacted-sperm or non reacted-sperm (without or with fluorescence in the acrosomal region, respectively).

#### Spermatozoa pH_i_

BCECF-AM stained and de-esterified spermatozoa were resuspended in TALP medium at pH 7.0, 7.4, 8.0 or 8.5 in presence or absence of OFCM. The fluorescence was monitored for 90s by spectrofluorimetry. The 490/440 nm ratio was recorded and the regression line for presence or absence of OFCM for all pH_e_ studied was calculated. The pH_i_ was determined from the regression lines in 4 replicates.

### Statistical Analysis

All statistical analyses were performed using SPSS v.20 (SPSS Inc. Chicago, IL, USA). Data are presented as mean ± SEM and were fitted to the binomial variable model. In Experiment 1, the rates of oocyte penetration, monospermy and efficiency were analyzed by three-way ANOVA, considering pH_e_, device and OFCM addition as the main variables. Efficiency was defined as the rate of monospermic oocytes with two pronuclei arising from the total number of inseminated oocytes. In Experiment 2.1, the data were analyzed by two-way ANOVA, considering pH_e_ and OFCM addition as the main variables. When ANOVA revealed a significant effect, values were compared using the least significant difference pairwise multiple comparison post hoc test (Tukey). In Experiment 2.2, regression lines were plotted and a non-parametric Kolmogórov-Smirnov test for normality of variables was applied. Mean values were analyzed and compared by Student’s t-test for two independent samples and correlation coefficients of Pearson were calculated. For all experiments, the level of significance was set at P < 0.05.

## Additional Information

**How to cite this article:** Soriano-Úbeda, C. *et al*. Improving porcine *in vitro* fertilization output by simulating the oviductal environment. *Sci. Rep.* **7**, 43616; doi: 10.1038/srep43616 (2017).

**Publisher's note:** Springer Nature remains neutral with regard to jurisdictional claims in published maps and institutional affiliations.

## Figures and Tables

**Table 1:**
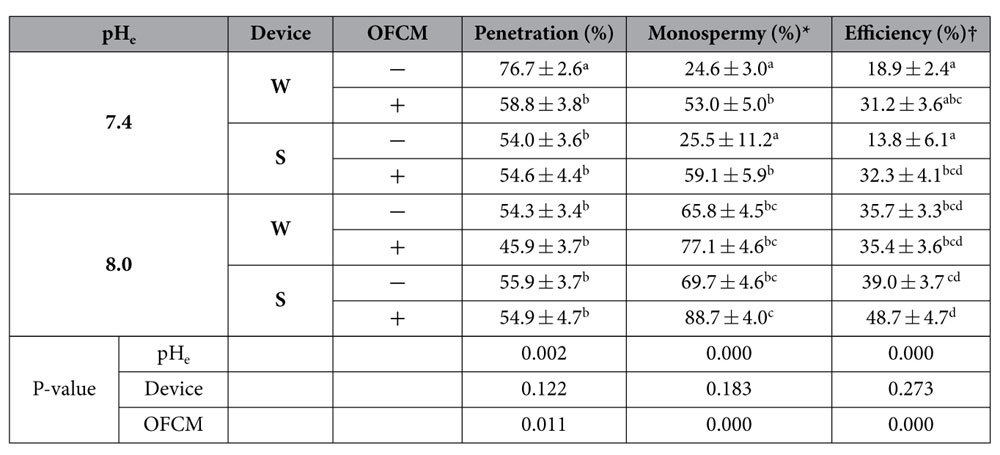
Effect of pH_e_, device and OFCM addition to TALP medium on pig IVF results.

The IVF was carried out at pH 7.4 or 8.0, in a 4-well multidish (W) or an inverted screw cap of a tube (S) and in presence or absence of OFCM (1% OF and 2% CM). Data are expressed as mean ± SEM. *From the penetrated oocytes. ^†^Efficiency was defined as the rate of monospermic oocytes with two pronuclei expressed as a percentage of the total number of inseminated oocytes.

Different (^a-d^) superscripts in the same column indicate significantly different values (P < 0.05).

**Table 2:**
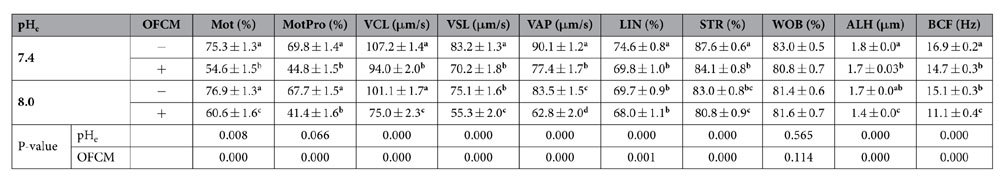
Effect of pH_e_ and OFCM addition on sperm motility parameters.

Spermatozoa were incubated in TALP medium for 30 min at pH 7.4 or 8.0 in presence or absence of OFCM (1% OF and 2% CM) and motility parameters were measured by CASA. Results are expressed as mean ± SEM.

Two-way ANOVA in which dependent variables were: Mot: percentage of total motile spermatozoa; MotPro: percentage of motile progressive spermatozoa; VCL: curvilinear velocity; VSL: straight-line velocity; VAP: average path velocity; LIN: linearity of the curvilinear trajectory (ratio VSL/VCL); STR: straightness (ratio VSL/VAP); WOB: Wobble (ratio VAP/VCL); ALH: amplitude of lateral head displacement; BCF: beat cross-frequency. Different superscripts (^a-c^) in the same column indicate statistically significant differences (P < 0.05).
